# Quality evaluation of Angelicae acutilobae radix: individual differences and localization of (*Z*)-ligustilide in *Angelica acutiloba* root

**DOI:** 10.1007/s11418-020-01438-1

**Published:** 2020-07-31

**Authors:** Yoshitomi Kudo, Hirokazu Ando, Yohei Sasaki

**Affiliations:** Laboratory of Molecular Pharmacognosy, Division of Pharmaceutical Sciences, Kanazawa University, Kakuma-machi, Kanazawa, Ishikawa 920-1192 Japan

**Keywords:** *Angelica acutiloba*, Angelicae acutilobae radix, (*Z*)-Ligustilide, Quality evaluation

## Abstract

It has been difficult to evaluate the quality of Angelicae acutilobae Radix (Toki) because of large differences in the contents of its chemical constituents. In this research, we revealed individual differences and localization of (*Z*)-ligustilide in Toki cultivated and processed under the same conditions. Thirteen Toki samples (dry weight: 68.2 g–132.3 g) were divided and categorized into 13 parts and the (*Z*)-ligustilide content of each part was quantified. Total (*Z*)-ligustilide content ranged from 0.08% to 0.22% and the maximum difference between samples was approximately 2.8-fold. In addition, the localization of (*Z*)-ligustilide was examined. (*Z*)-Ligustilide content was the highest in lateral root at 0.19%, followed by main root at 0.13%, and the lowest in root head at 0.09%. Furthermore, the content tended to increase as the root became thinner. In particular, the difference in content between the inner side of upper root head (removed 5 mm from the epidermis 0.06%) and the 1.1–3.0 mm in diameter lateral root (0.24%) was largest at approximately 4.1-fold. We revealed that not only differences among individuals but also localization is a factor affecting the quality of Toki. In contrast, individuals with higher root part (main root + lateral root) weight ratio in whole root dry weight had higher (*Z*)-ligustilide content. The difference in (*Z*)-ligustilide content among individuals is due to the balance between root head part and root other than head part. It is possible to predict (*Z*)-ligustilide content from weight ratio of root part to whole root.

## Introduction

*Angelica acutiloba* (Siebold et Zucc.) Kitag. (Umbelliferae) roots are used as the herbal medicine “Toki (当帰)” and blended into such Kampo formulas as Shimotsuto and Tokisyakuyakusan. Although most herbal medicines used in Japan are dependent on imports from overseas, Toki is one of the most important medicinal plants that have relatively high self-sufficiency in Japan [1].

Since ancient times, the quality of Toki has been evaluated on the basis of taste, odor, and shape. Toki that is sweet, pungent, and fragrant is said to be of high quality [2]. Furthermore, Toki with lateral roots bundled like a horse tail is also considered to be of good quality and called “Babi Toki (馬尾当帰) [3] ”, which means Toki shaped like horse tail. It is understood from the illustration inserted in the reference no. 3 that Babi Toki has a high proportion of lateral roots. It has been reported that lateral roots of (*Z*)-ligustilide are higher than those of main roots and fine roots in *Angelica acutiloba* root [4, 5]. This suggests that the quality of Toki is affected by ratio of lateral roots. In addition, the shape of Toki is defined as follows in the Japanese Pharmacopoeia Seventeenth Edition (JP17): “Thick and short main root, with numerous branched roots, nearly fusiform, externally dark brown to red-brown, with longitudinal wrinkles and horizontal protrusions composed of numerous scars of fine rootlets” [6]. It is thought that the higher the ratio of lateral roots to the whole roots, the better the medicinal quality of Toki based on those references [3, 6].

At present, quality is evaluated by chemical methods in addition to conventional quality evaluation methods, such as taste, odor, and shape evaluation. In JP17, a dilute ethanol-soluble extract of Toki is required not less than 35% [6]. In addition, (*Z*)-ligustilide, one of the major essential oils in Toki, is reported to be related to not only the fragrance of Toki [7] but also its anti-acetylcholine action [8] and vasodilatory action [9], and is frequently used as a quality evaluation indicator [10–15].

We have been conducting research of Angelicae acutilobae radix production at the Medicinal Plant Garden of the School of Pharmacy, School of Pharmaceutical Sciences, Kanazawa University. However, because of large individual differences in (*Z*)-ligustilide content, it is not possible to evaluate differences due to experimental conditions, such as processing temperature and time. To study the quality of Toki, it is necessary to clarify the cause of individual differences in (*Z*)-ligustilide content. It has been reported that lateral roots contained more (*Z*)-ligustilide than main roots and fine roots [4, 5], but the experimental material used in these reports was small (less than 20 g dry weight). Toki used in medicine is at least larger than 50 g, and the “root part” grows at such a size of root. In China, Chinese-Toki sometimes uses only the root head named “Toki head (帰頭)” and the lateral root named “Toki tail (帰尾)” [16]. This fact suggests a difference in the medical efficacy depending on the part in the root of Toki. Therefore, the purpose of this study is to distinguish roots into main roots, lateral roots, and root heads, and to verify the content of (*Z*)-ligustilide as the main component. In this paper, roots were divided into small parts, and the content of (*Z*)-ligustilide was measured.

Toki produced at the medicinal plant garden in Kanazawa University is of a quality that is used in clinical. In this study, Toki produced under the same cultivation and processing conditions was categorized into 13 parts in more detail than in previous studies and the shape was evaluated after measuring the weight of each part. In addition, we investigated the localization of (*Z*)-ligustilide and the content difference between individuals by quantifying (*Z*)-ligustilide in different root parts. Finally, the correlation between the weight or weight ratio of each root part and (*Z*)-ligustilide content was determined, it was examined whether individuals with a high ratio of lateral roots had a high (*Z*)-ligustilide content in whole root.

## Materials and methods

### Materials and sample processing

One-year-old plants that were seeded at the Medicinal Plant Garden of the School of Pharmacy, School of Pharmaceutical Sciences, Kanazawa University on April 19, 2016 were planted on April 15, 2017, cultivated for approximately 7 months, and harvested on November 14, 2017. They were air-dried outdoors until December 23 and then removed from the ground and washed with water.

**Fig. 1:**
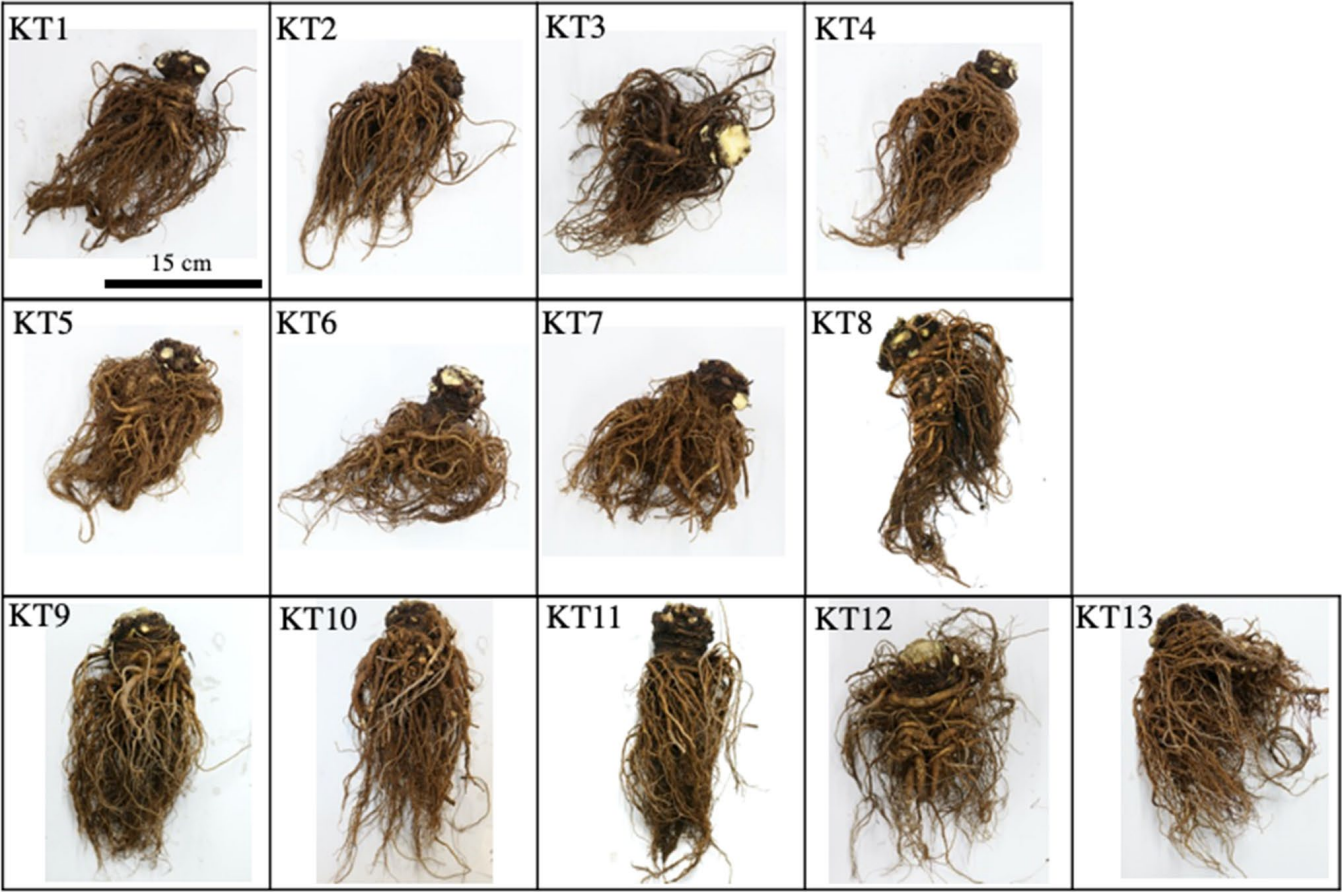
*A. acutiloba* roots used in experiment. After harvest, the roots were air dried from November 14 to December 23, 2017 and then washed with water

**Fig. 2:**
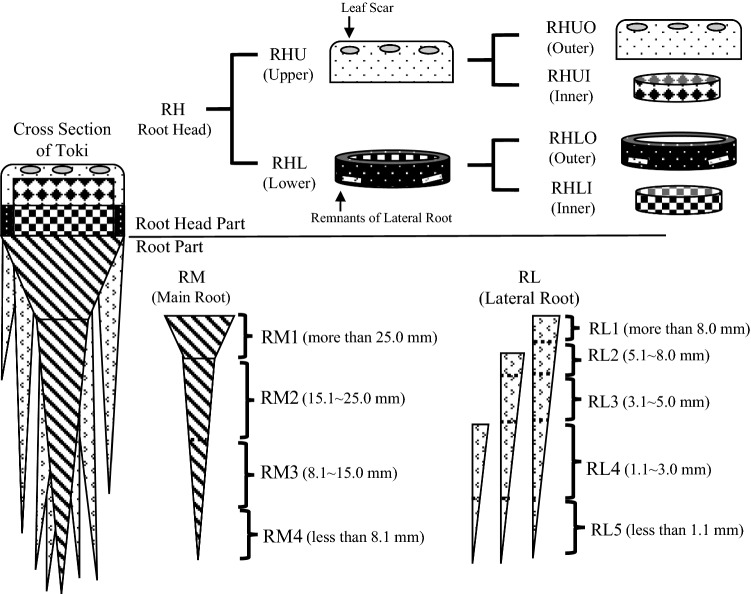
Cutting and classification method of *A. acutiloba* roots. Each individual was divided into 13 parts (RHUO, RHUI, RHLO, RHLI, RM1, RM2, RM3, RM4, RL1, RL2, RL3, RL4, RL5)

**Fig. 3:**
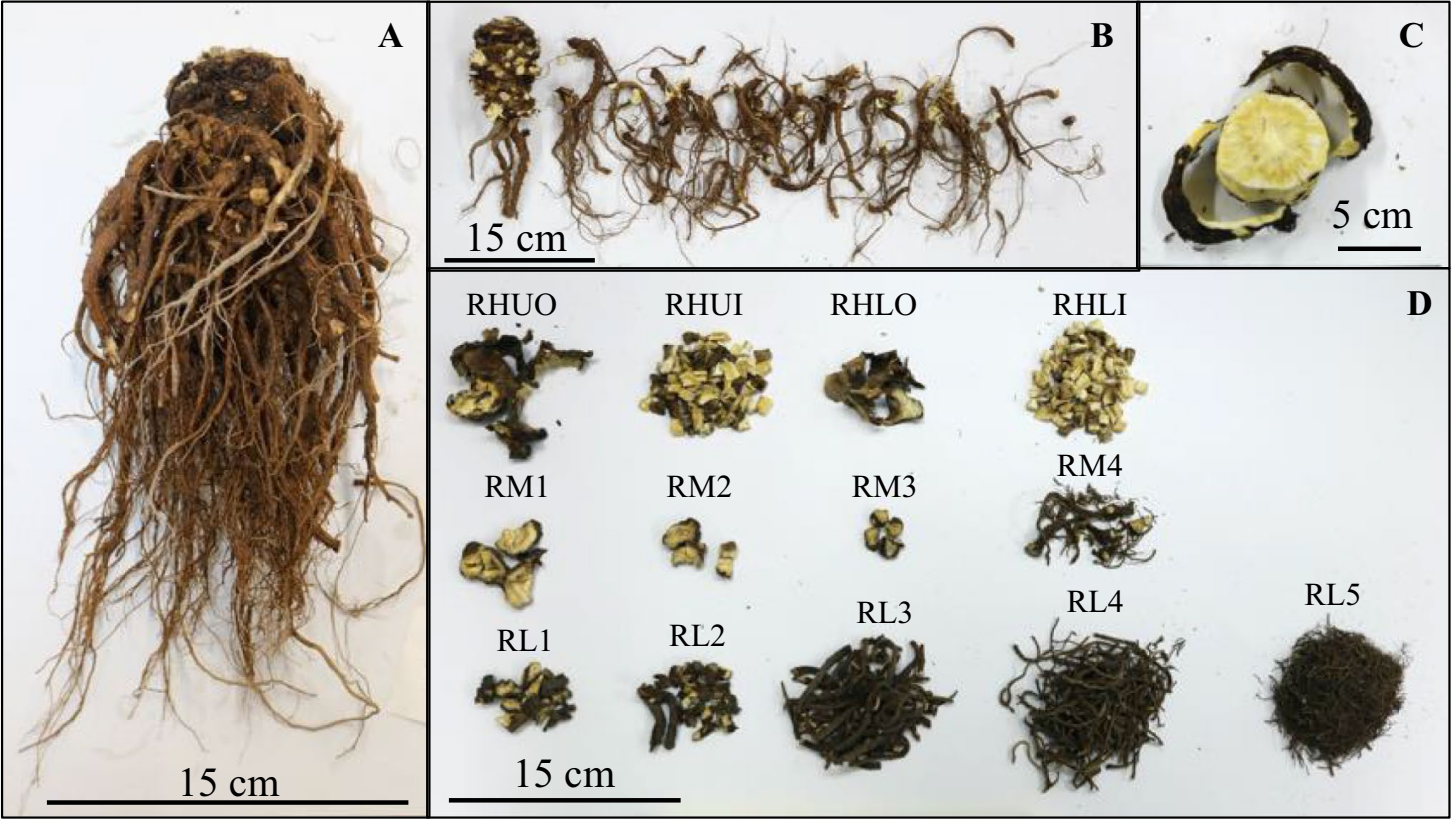
**a** *A. acutiloba* roots after natural drying (Sample: KT10). **b** Separating lateral roots from whole roots. **c** Separating the outside from upper root head. **d** All samples prepared from dried *A. acutiloba* roots

Thirteen Toki samples (KT1-13, Fig. 1) were divided into 13 parts (RHUO, RHUI, RHLO, RHLI, RM1, RM2, RM3, RM4, RL1, RL2, RL3, RL4, RL5), as described below (Figs. 2 and 3). First, the root was divided into three parts (root head, main root, and lateral root). The root head is the rhizome and upper root, from the border with the above-ground part to the part where the first lateral root occurs. The main root was the root that extended directly downward from the root head, and the lateral root was the other roots. The root head was divided into the upper part that includes the leaf scar and the lower part that does not include it. Furthermore, the upper part was divided into two parts: the outer side that is 5 mm from the epidermis (RHUO) and the inner side (RHUI). The lower part was also divided into two parts in the same way as the upper part: the outer side (RHLO) and the inner side (RHLI).

Each part was dried at 60 °C for 12 h using a constant-temperature dryer (ETTAS, EOP-450B), left at room temperature for approximately 12 h, and dried again at 60 °C for 12 h using the constant-temperature dryer. After that, the main root was classified into four grades (RM1: more than 25.0 mm in diameter, RM2: 15.1–25.0 mm in diameter, RM3: 8.1–15.0 mm in diameter, RM4: less than 8.1 mm in diameter) according to diameter and the lateral root was classified into five grades (RL1: more than 8.0 mm in diameter, RL2: 5.1–8.0 mm in diameter, RL3: 3.1–5.0 mm in diameter, RL4: 1.1–3.0 mm in diameter, RL5: less than 1.1 mm in diameter), and each sample was obtained. After measuring the weight of each sample, the sample was ground and used in subsequent experiments.

The weight and the (*Z*)-ligustilide content of total root head (RH) were calculated from the weights and contents of RHUO, RHUI, RHLO, and RHLI. The weight and the (*Z*)-ligustilide content of whole main root (RM) were calculated from the weights and contents of RM1, RM2, RM3, and RM4. The weight and the (*Z*)-ligustilide content of total lateral root (RL) were calculated from the weights and contents of RL1, RL2, RL3, RL4, and RL5. A commercial Toki product (Tochimoto Tenkaido Co., Ltd., Lot no.: 008014005) was used for comparison.

### Quantification of (*Z*)-ligustilide

To an accurately weighed sample (0.03 g), 10 ml of MeOH was added, and this was followed by ultrasonic extraction for 30 min under room temperature. The extract was centrifuged for 10 min at 13,000 rpm and the supernatant was passed through a membrane filter (FILTSTAR 0.45 µm Nippon Genetics) to prepare the sample solution for HPLC. Subsequently, (*Z*)-ligustilide was measured by HPLC. Total (*Z*)-ligustilide content (= (*Z*)-ligustilide content per sample) was calculated from the (*Z*)-ligustilide content and the dry weight of each part.

In this experiment, the drying process was performed immediately prior to sample preparation and was not corrected for loss on drying.

### HPLC conditions

A Hitachi L-2200 high-performance liquid chromatograph and a COSMOSIL 5C18-MS-II Packed Column (*φ* 4.6 × 250 mm) were used under the following conditions: mobile phase: methanol/1% acetic acid (7:3), column temperature: 40 °C, flow rate: 1.0 ml/min, UV detection: 320 nm, and injection volume: 10 µl. The standard was (*Z*)-ligustilide standard solution (0.1 mg/ml methanol solution; Wako Pure Chemical Industries, TWH1363). Calibration curve was *Y* = 24,900,886.651 *X* − 9,247.998, *R*^2^ = 0.99999.

### Correlation between weight or weight ratio of each root part and total (*Z*)-ligustilide content

For root head (RH), main root (RM), lateral root (RL), and root (RM + RL), Pearson’s correlation coefficient between weight or weight ratio and total (*Z*)-ligustilide content was investigated.

In general, Toki undergoes processing, such as Hasagake (natural drying) and Yumomi (hot water treatment) [17], so most of the thin roots (less than 1 mm in diameter) fall off. Therefore, the correlation was also investigated when lateral root (RL) measuring less than 1.1 mm in diameter was removed from the root as the processing condition (RM + RL − RL5).

## Results

### Evaluation of root weight or weight ratio of each root part

The dry weights of whole root and different root parts of each sample are shown in Table 1. Whole root dry weights of the 13 individuals were 68.2–132.3 g (average 96.0 ± 5.6 g). The weights of root head (RH) were 22.2–58.0 g (average 32.8 ± 2.9 g), those of main root (RM) were 10.4–33.0 g (average 18.2 ± 1.8 g), and those of lateral root (RL) were 28.6–69.7 g (average 45.0 ± 2.9 g). The weight ratio of each part to whole root dry weight was 29.0–43.8% (average 33.8 ± 1.4%) for root head (RH), 10.6–25.4% (average 19.0 ± 1.4%) for main root (RM), and 31.3–56.6% (47.2 ± 2.0%) for lateral root (RL). These results indicated large differences in weight ratio of each root part among individuals.

**Table 1:** Dry weights of whole root and different parts of samples

Sample	RHUI	RHUO	RHLI	RHLO	Total RH	RM1	RM2	RM3
1	6.6	8.2	4.0	3.4	22.2	–	5.8	1.8
2	11.6	10.2	5.5	3.0	30.3	3.9	2.0	1.8
3	9.5	5.5	5.4	3.2	23.5	–	5.2	2.2
4	7.1	6.7	5.7	4.3	23.8	–	6.7	1.2
5	5.7	6.0	9.4	5.4	26.5	5.3	1.9	0.9
6	7.9	11.9	6.4	3.2	29.3	11.5	1.4	2.7
7	7.9	9.6	6.5	2.7	26.7	7.0	3.9	4.0
8	11.9	9.3	5.4	2.7	29.3	9.2	5.7	1.9
9	13.9	7.9	11.1	2.8	35.6	7.1	3.9	1.7
10	10.2	6.4	9.4	4.9	30.9	15.3	1.5	1.4
11	19.9	10.6	12.8	3.0	46.4	9.7	3.2	1.3
12	12.7	5.9	20.3	5.7	44.5	4.1	–	2.4
13	17.5	12.9	18.7	8.9	58.0	–	7.1	4.7
Ave.	11.0	8.5	9.3	4.1	32.8	8.1	4.0	2.2
S.D.	4.1	2.3	5.0	1.7	10.2	3.5	2.0	1.0

Sample	RM4	Total RM	RL1	RL2	RL3	RL4	RL5	Total RL	Whole root
1	9.8	17.4	2.2	3.4	8.0	9.9	5.1	28.6	68.2
2	6.9	14.7	–	1.9	6.8	15.9	4.5	29.1	74.1
3	3.1	10.4	4.6	5.2	13.9	15.2	5.4	44.3	78.2
4	2.8	10.7	–	1.5	21.8	14.4	6.6	44.3	78.8
5	6.0	14.0	4.4	8.2	10.0	12.3	7.0	41.9	82.5
6	4.4	20.0	1.6	6.9	12.8	12.7	6.2	40.1	89.5
7	5.0	19.8	3.1	12.2	15.3	9.3	6.9	46.8	93.3
8	7.0	23.9	10.8	6.3	12.0	12.9	5.6	47.6	100.8
9	3.8	16.5	6.9	7.5	18.2	11.1	8.0	51.7	103.8
10	7.8	26.0	3.7	11.2	24.0	9.7	2.9	51.6	108.5
11	2.3	16.5	9.8	5.5	14.6	12.8	5.5	48.2	111.0
12	7.1	13.6	4.0	20.3	23.5	14.2	7.6	69.7	127.8
13	21.2	33.0	2.3	16.0	7.6	8.0	7.5	41.4	132.3
Ave.	6.7	18.2	4.9	8.2	14.5	12.2	6.1	45.0	96.0
S.D.	4.7	6.1	2.9	5.3	5.7	2.3	1.4	9.9	19.4

*RH* root head, *RM* main root, *RL* lateral root, *RHUI* inner side of upper root head, *RHUO* outer side of upper root head, *RHLI* inner side of lower root head, *RHLO* outer side of lower root head, Total RH = RHUI + RHUO + RHLI + RHLO, *RM1* main root measuring more than 25.0 mm in diameter, *RM2* main root measuring 15.1–25.0 mm in diameter, *RM3* main root measuring 8.1–15.0 mm in diameter, *RM4* main root measuring less than 8.1 mm in diameter, Total RM = RM1 + RM2 + RM3 + RM4, *RL1* lateral root measuring more than 8.0 mm in diameter, *RL2* lateral root measuring 5.1–8.0 mm in diameter, *RL3* lateral root measuring 3.1–5.0 mm in diameter, *RL4* lateral root measuring 1.1–3.0 mm in diameter, *RL5* lateral root measuring less than 1.1 mm in diameter, Total RL = RL1 + RL2 + RL3 + RL4 + RL5, whole root = Total RH + Total RM + Total RL, “–” indicates no sample available

### Inter-individual differences in dry weight and total (*Z*)-ligustilide content

**Fig. 4:**
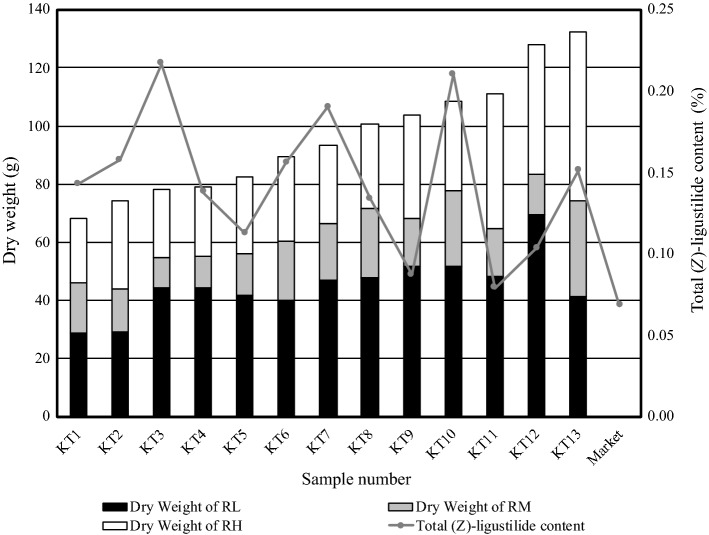
Inter-individual differences in dry weight and total (*Z*)-ligustilide content in whole root. Total (*Z*)-ligustilide content was calculated from (*Z*)-ligustilide content and dry weight of each part. *RH* root head, *RM* main root, *RL* lateral root

The 13 individuals were arranged in an increasing order of dry weight (Fig. 4). Total (*Z*)-ligustilide content was lowest at 0.08% for KT11 and highest at 0.22% for KT3 (average 0.14 ± 0.01%). Inter-individual difference in content was as high as approximately 2.7-fold. (*Z*)-ligustilide content of commercial Toki product was 0.07%.

### (*Z*)-ligustilide content in different parts of *A. acutiloba* root

**Fig. 5:**
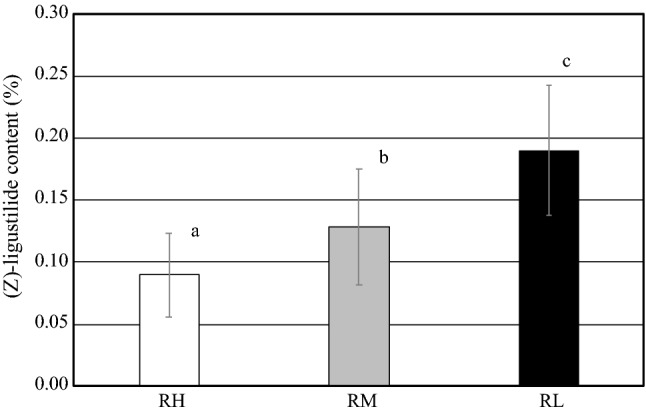
(*Z*)-Ligustilide content in different parts of *A. acutiloba* root. Lowercase letters indicate significant difference between different characters evaluated by Student’s *t* test with Bonferroni correction (*p* < 0.01). Data are shown as mean ± S.D. (*n* = 13). *RH* root head, *RM* main root, *RL* lateral root

First, the (*Z*)-ligustilide contents in root head (RH), main root (RM), and lateral root (RL) were compared (Fig. 5). (*Z*)-Ligustilide content in lateral root (RL) was highest at 0.12–0.27% (average 0.19 ± 0.02%), followed by that in main root (RM) at 0.07–0.24% (average 0.13 ± 0.01%) and that in root head (RH) at 0.03–0.14% (average 0.09 ± 0.01%). There were significant differences between all pars (*p* < 0.01, Student’s *t* test with Bonferroni correction).

**Fig. 6:**
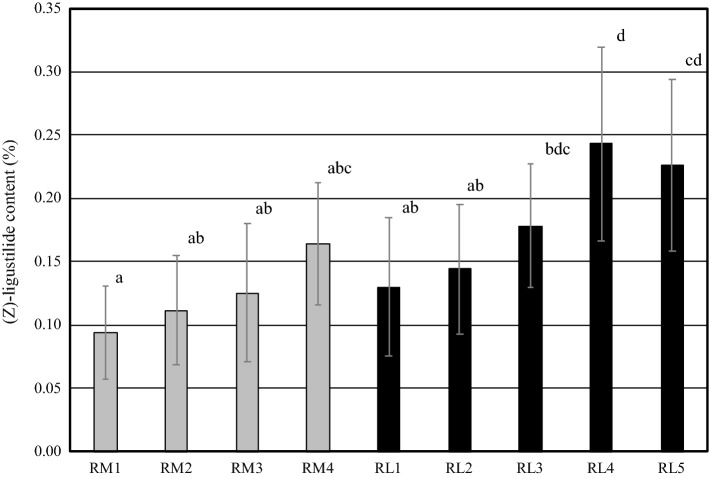
(*Z*)-Ligustilide content in main root and lateral root having different root diameters. Lowercase letters indicate significant difference between different characters as evaluated by Tukey’s multiple comparison test (*p* < 0.05). Data are shown as mean ± S.D. (*n* = 9–13). *RM* main root, *RM1* more than 25.0 mm in diameter, *RM2* 15.1–25.0 mm in diameter, *RM3* 8.1–15.0 mm in diameter, *RM4* less than 8.1 mm in diameter, *RL* lateral root, *RL1* more than 8.0 mm in diameter, *RL2* 5.1–8.0 mm in diameter, *RL3* 3.1–5.0 mm in diameter, *RL4* 1.1–3.0 mm in diameter, *RL5* less than 1.1 mm in diameter

Next, (*Z*)-ligustilide contents in main root (RM) were compared by root diameter. (*Z*)-ligustilide content was 0.04–0.16% (average 0.09 ± 0.01%) in RM1 measuring more than 25 mm in diameter; 0.07–0.24% (average 0.11 ± 0.01%) in RM2 measuring 15.1–25.0 mm in diameter; 0.07–0.24% (average 0.13 ± 0.01%) in RM3 measuring 8.1–15.0 mm in diameter; and 0.09–0.26% (average 0.16 ± 0.02%) in RM4 measuring less than 8.1 mm in diameter. The content tended to increase as the root became thinner (Fig. 6).

Next, (*Z*)-ligustilide contents in lateral root (RL) were 0.06–0.25% (average 0.13 ± 0.02%) in RL1 measuring more than 8 mm in diameter; 0.07–0.26% (average 0.14 ± 0.02%) in RL2 measuring 5.1–8.0 mm in diameter; 0.10–0.25% (average 0.18 ± 0.01%) in RL3 measuring 3.1–5.0 mm in diameter; 0.15–0.41% (average 0.24 ± 0.02%) in RL4 measuring 1.1–3.0 mm in diameter; and 0.13–0.36% (average 0.23 ± 0.02%) in RL5 measuring less than 1.1 mm in diameter. As in the case of main root (RM), the content increased as the roots became thinner (Fig. 6).

**Fig. 7:**
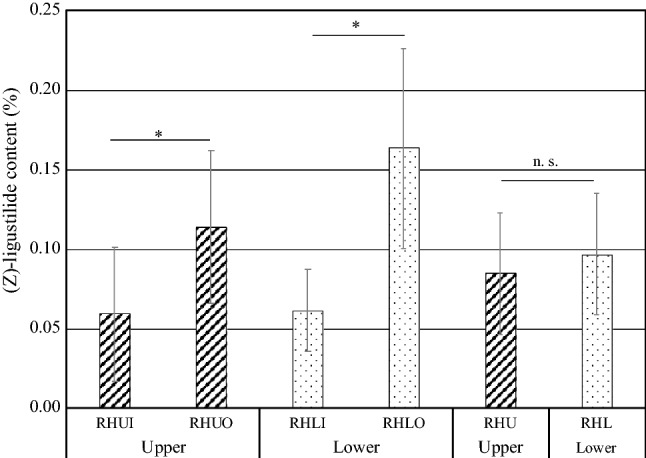
(Z)-Ligustilide content in different parts of root head. *p < 0.01, n.s. means not significant as evaluated by the Student’s *t* test. *RHUI* inner side of upper root head, *RHUO* outer side of upper root head, *RHLI* inner side of lower root head, *RHLO* outer side of lower root head, *RHU* upper root head, *RHL* lower root head. RHUI compared with RHUO. RHLI compared with RHLO. RHU compared with RHL. Data are shown as mean ± S.D. (*n* = 13)

Finally, (*Z*)-ligustilide contents were compared in different parts of root head (RH) (Fig. 7). There was no significant difference in (*Z*)-ligustilide content between upper root head (RHU, 0.08%) and lower root head (RHL, 0.1%, *p* > 0.05, Student’s *t* test). On the other hand, (*Z*)-ligustilide contents in both upper and lower root heads were significantly higher on the outer side than on the inner side (RHUI: 0.06% v.s. RHUO: 0.11% *p* < 0.01, RHLI: 0.06% v. s. RHLO: 0.16% *p* < 0.01, Student’s *t* test).

### Correlation between weight or weight ratio of each root part and total (*Z*)-ligustilide content

**Fig. 8:**
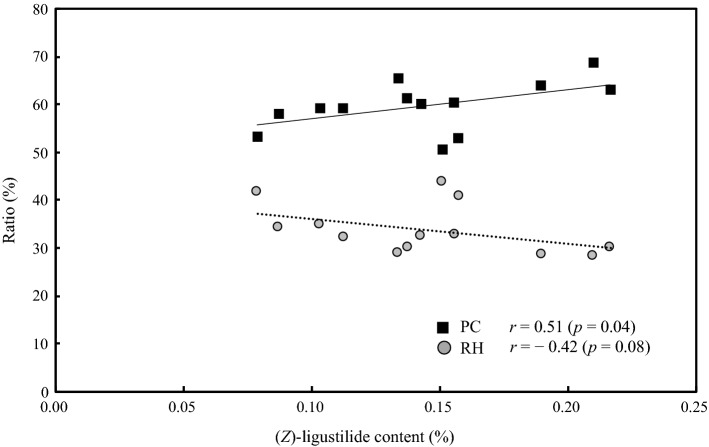
Correlation between weight ratio of each part to whole root dry weight and total (*Z*)-ligustilide content. *RH* root head, *PC* processing condition (RM + RL − RL5), *RM* main root, *RL* lateral root, *RL5* lateral root less than 1.1 mm in diameter. *r*: Pearson’s correlation coefficient. PC is significant in 5% level

The correlation between root shape and total (*Z*)-ligustilide content was investigated (Fig. 8). The correlation coefficients between total (*Z*)-ligustilide content and the weight of each part were as follows: root head (RH; *r* = − 0.40, *p* = 0.09), main root (RM; *r* = 0.17, *p* = 0.30), lateral root (RL; *r* = − 0.26, *p* = 0.20, root (RM + RL; *r* = − 0.13, *p* = 0.33), and processing condition (RM + RL − RL5; *r* = − 0.07, *p* = 0.41); there is no significant correlation at 5% level, but relatively high negative correlation was observed between total (*Z*)-ligustilide content and the weight of RH. On the other hand, the correlation coefficients between the weight ratio of each part to whole root dry weight and total (*Z*)-ligustilide content were as follows: root head (RH; *r* = − 0.42, *p* = 0.80) (Fig. 8), main root (RM; *r* = 0.34, *p* = 0.13), lateral root (RL; *r* = 0.07, *p* = 0.41), root (RM + RL; *r* = 0.42, *p* = 0.08), and processing condition (RM + RL − RL5; *r* = 0.51, *p* = 0.04) (Fig. 8). Relatively strong negative correlation was observed between the weight ratio of root head and total (*Z*)-ligustilide content. And there is a significant correlation in 5% level was observed between the weight ratio of processing condition and total (*Z*)-ligustilide. On the other hand, there was no correlation between whole root weight and total (*Z*)-ligustilide content (*r* = − 0.29, *p* = 0.17).

## Discussion

### Inter-individual differences (whole root dry weight, root shape, total (*Z*)-ligustilide content)

There was no clear correlation between whole root dry weight and the weight ratio of each part to whole root dry weight among the 13 individuals. In addition, total (*Z*)-ligustilide content was 0.08–0.22%, and there was a maximum difference of approximately 2.8-fold between individuals. In this study, Toki individuals grown under the same cultivation and processing conditions were used in the experiments, but the produced Toki showed large differences in whole root dry weight, weight of each root part, weight ratio of each root part, and (*Z*)-ligustilide content.

In other medicinal plants, there were reports that the morphological characteristics and the content and composition of chemical constituents differ among individuals. Differences among such individuals are genetically controlled, and differences are smaller for genetically equal individuals [18]. In general, Toki proliferates by seed breeding, so its morphological characteristics or chemical constituents tend to differ easily among individuals. The differences among individuals observed in this study are thought to originate from genetic factors. From the above results, it is considered that the effects of individual differences in root weight ratio of each root part and (*Z*)-ligustilide content cannot be ignored in quality evaluation.

### (*Z*)-Ligustilide distribution in different parts of *A. acutiloba* root

Within the same individual, (*Z*)-ligustilide content was highest (average 0.24%) in lateral root measuring 1.1–3.0 mm in diameter (RL4) and lowest (average 0.06%) in the upper inner side of root head (RHUI), and the difference in (*Z*)-ligustilide content among different root parts was approximately 4.0-fold. It was already reported that (*Z*)-ligustilide content was highest in the lateral roots and lowest in the fibrous root, and this study showed similar result [4, 5]. Even when classified into root, main root, and lateral root, the difference in (*Z*)-ligustilide content between lateral root (RL, 0.19%) and root head (RH, 0.09%) was approximately 2.1-fold. In other words, the localization of (*Z*)-ligustilide in the same individual may have a greater impact on quality evaluation than individual differences. In addition, it was revealed that root head contained more (*Z*)-ligustilide on the outside than the inside. In several medicinal plants that use roots as crude drugs, important chemical constituents for quality evaluation are localized in tissues near the root surface [19–21]. Similarly, in Toki, (*Z*)-ligustilide may be localized in tissues near the root surface. Strong insecticidal activity has been observed in (*Z*)-ligustilide in the Chinese medicinal plant *Ligusticum chuanxiong* [22] and antifungal activity has been reported in essential oils of other plants [23–25]. Therefore, (*Z*)-ligustilide in Toki may function as a defense mechanism against insects and fungi by localizing near the root surface.

Furthermore, it was clarified that (*Z*)-ligustilide content increased as the root became thinner in both main and lateral roots. This was probably because the weight of tissue near the epidermis increased as the root became thinner. Previous studies reported that saikosaponins contained in *Bupleurum falcatum* is abundant in the outer phloem layer, especially the pericycle and its neighbouring parenchyma cells in the root, and highest level of saikosaponins was detected in thinner root hairs and that the level decreased towards the thicker root head. This difference in saikosaponins content is attributed to the proportion of root tissue, and it has also been reported that the proportion of phloem increases with thinner roots [26]. Similarly, in Toki, it is presumed that as the roots became thinner, the proportion of relatively outer tissues such as the phloem, pericycle and its neighbouring parenchyma increased, and the (*Z*)-ligustilide content increased.

In some individuals, (*Z*)-ligustilide content was higher in the 1.1- to 3.0-mm-diameter lateral root (RL4) than in the thinnest lateral root (RL5) of less than 1.1 mm in diameter. This indicates that (*Z*)-ligustilide might have flowed out and decomposed in lateral roots measuring 1 mm or less in diameter due to the influence of washing and drying during sample processing. It is also possible that the tissue was not fully developed and secretion and accumulation were insufficient.

Total (*Z*)-ligustilide content of commercial Toki product is 0.07%, which is the lowest compared with those of the thirteen individuals examined in this study. The reason may be that thin roots having high (*Z*)-ligustilide content were lost during processing. Furthermore, it is surmised that (*Z*)-ligustilide volatilized because root surface area was increased by cutting.

### Correlation between weight or weight ratio of each root part and total (*Z*)-ligustilide content

When the correlations between total weight, weight of different root parts or weight ratio of each part to whole root dry weight and total (*Z*)-ligustilide content were investigated, no correlation was found between total weight and total (*Z*)-ligustilide content. Therefore, it was difficult to predict total (*Z*)-ligustilide content from the total weight. Regarding the correlation between weight of different root parts and total (*Z*)-ligustilide content, a relatively strong negative correlation (*r* = − 0.40, *p* = 0.09) was observed for root head (RH), and the smaller the root head, the higher the content. With regard to the correlation between the weight ratio of each part to whole root dry weight and total (*Z*)-ligustilide content, the strongest negative correlation (*r* = − 0.42, *p* = 0.80) was observed for root head (RH) and a relatively strong positive correlation (*r* = 0.42, *p* = 0.08) was observed for root (RM + RL). Total (*Z*)-ligustilide content tedded to increase as the proportion of root increased. Furthermore, the strongest and significant positive correlation in 5% level (*r* = 0.51, *p* = 0.04) was observed between total (*Z*)-ligustilide content and the processing condition wherein lateral roots less than 1.1 mm in diameter were removed from the root part (RM + RL − RL5). In other words, total (*Z*)-ligustilide content was higher in individuals with higher root part (RM + RL − RL5) ratio. This correlation is related to the fact that lateral root has high (*Z*)-ligustilide content. The difference in content between individuals is thought to be influenced by the localization of (*Z*)-ligustilide.

*A*. *acutiloba* roots that are processed into herbal medicine undergo various processing steps, including Hasagake and Yumomi [17]. As a result, extremely thin roots measuring less than 1 mm in diameter fall off and are not used for Kampo formulas. In addition, roots measuring less than 1 mm in diameter have a larger surface area than thick roots, and may have been affected in various ways during sample preparation. Therefore, it is necessary to evaluate roots measuring less than 1 mm in diameter in the quality evaluation of Toki.

As described above, we have revealed that individuals with smaller root head and larger root part ratio (RM + RL − RL5) have higher (*Z*)-ligustilide contents, and the approximate content can be predicted from root part ratios (RM + RL − RL5). In other words, in quality evaluation, the effect of individual differences on the contents of chemical constituents can be reduced by making the shapes uniform. Conventionally, taste, odor, and shape have been regarded as important in the quality evaluation of Toki, and in this study, it has become clear that root part ratio (RM + RL − RL5) can affect quality evaluation. Toki with smaller root head and many root part (RM + RL − RL5) is considered to have the shape of ancient “Babi Toki” and has proven the usefulness of quality evaluation by their dry weight ratio to whole root dry weight.

It is empirically known that the same herbal medicine has different contents of chemical constituents depending on the part. However, no detailed investigation has been conducted regarding the effect of such on quality evaluation. To resolve this issue, the localization of chemical constituents has been investigated in several medicinal plants. In fact, the localization of components in root part was studied about *Paeonia moutan, Panax ginseng*, *Scutellaria baicalensis* and *Bupleurum falcatum*, and those major components were clarified [19–21, 26].

In conclusion, we revealed that (*Z*)-ligustilide, the main chemical constituent in Toki, was localized in different root parts in varying amounts. We also clarified that there was a large difference in (*Z*)-ligustilide content even if whole root weights were similar. Moreover, (*Z*)-ligustilide tended to localize in main roots and lateral roots. Therefore, individuals with higher root ratio had higher (*Z*)-ligustilide content, and a positive correlation was observed between root ratio (RM + RL − RL5) and (*Z*)-ligustilide content.

In this study, we clarified the individual differences in (*Z*)-ligustilide content by investigating the localization of chemical constituents in Toki. Our findings are expected to contribute to check the processing condition of Toki like temperature and time, which has been difficult so far because of the individual differences and the localization.
